# Supplementary material for: Regioselective Cyclic Iminium Formation of Ugi Advanced Intermediates: Rapid Access to 3,4-Dihydropyrazin-2(1H)-ones and Other Diverse Nitrogen-Containing Heterocycles
Source: Molecules. 2023 Mar 29;28(7):3062. doi: 10.3390/molecules28073062 (PMC10095709; doi:10.3390/molecules28073062)

Regioselective Cyclic Iminium Formation of Ugi Advanced Intermediates: Rapid Access to 3,4-Dihydropyrazin-2(1*H*)-ones and Other Diverse Nitrogen-Containing Heterocycles

Naděžda Cankařová [1,*] and Viktor Krchňák [1,2]

[1] Department of Organic Chemistry, Faculty of Science, Palacký University, 17 listopadu 12, 771 46 Olomouc, Czech Republic; viktor.krchnak.1@nd.edu
[2] Department of Chemistry and Biochemistry, 251 Nieuwland Science Center, University of Notre Dame, Notre Dame, IN 46556, USA
* Correspondence: nadezda.cankarova@upol.cz

Table of Contents

1. ^{1}H and ^{13}C NMR Spectra S2

1. ^{1}H and ^{13}C NMR Spectra

^{1}H and ^{13}C NMR spectra (d_6-DMSO) for compound 2a

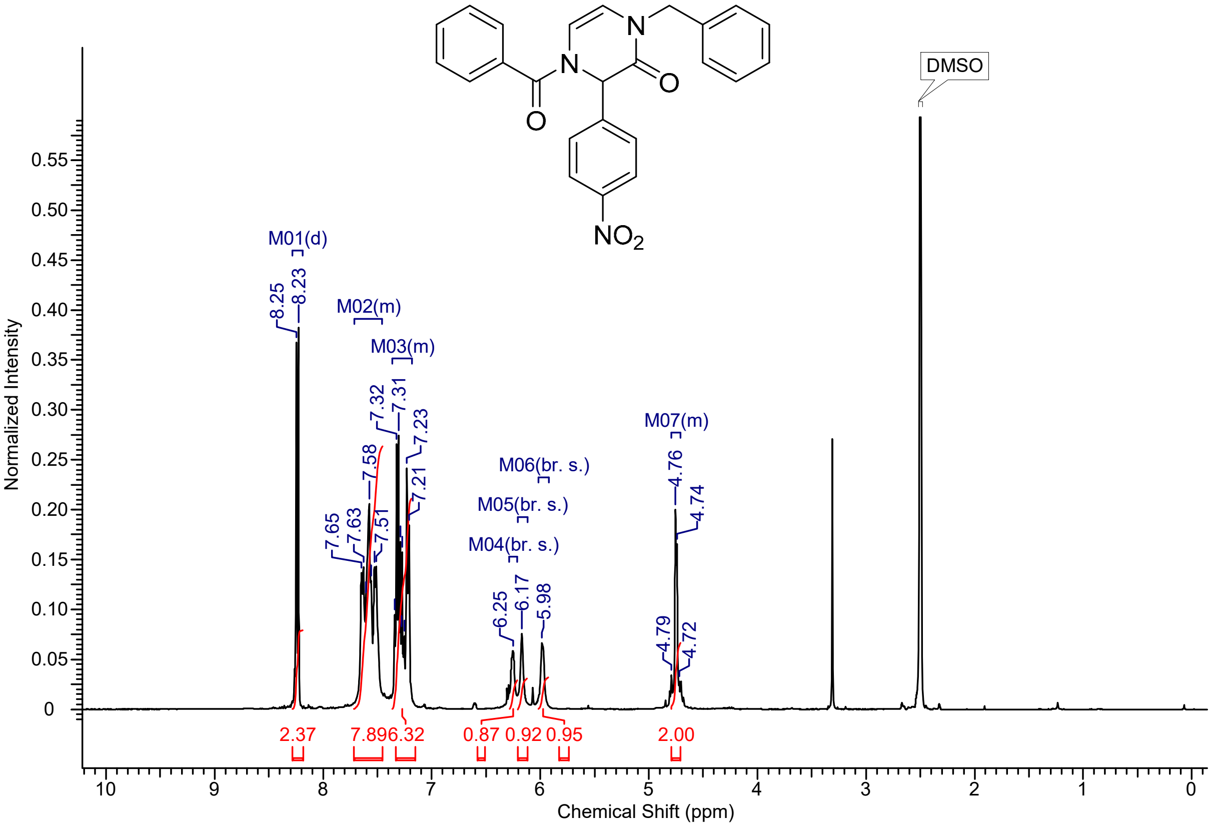

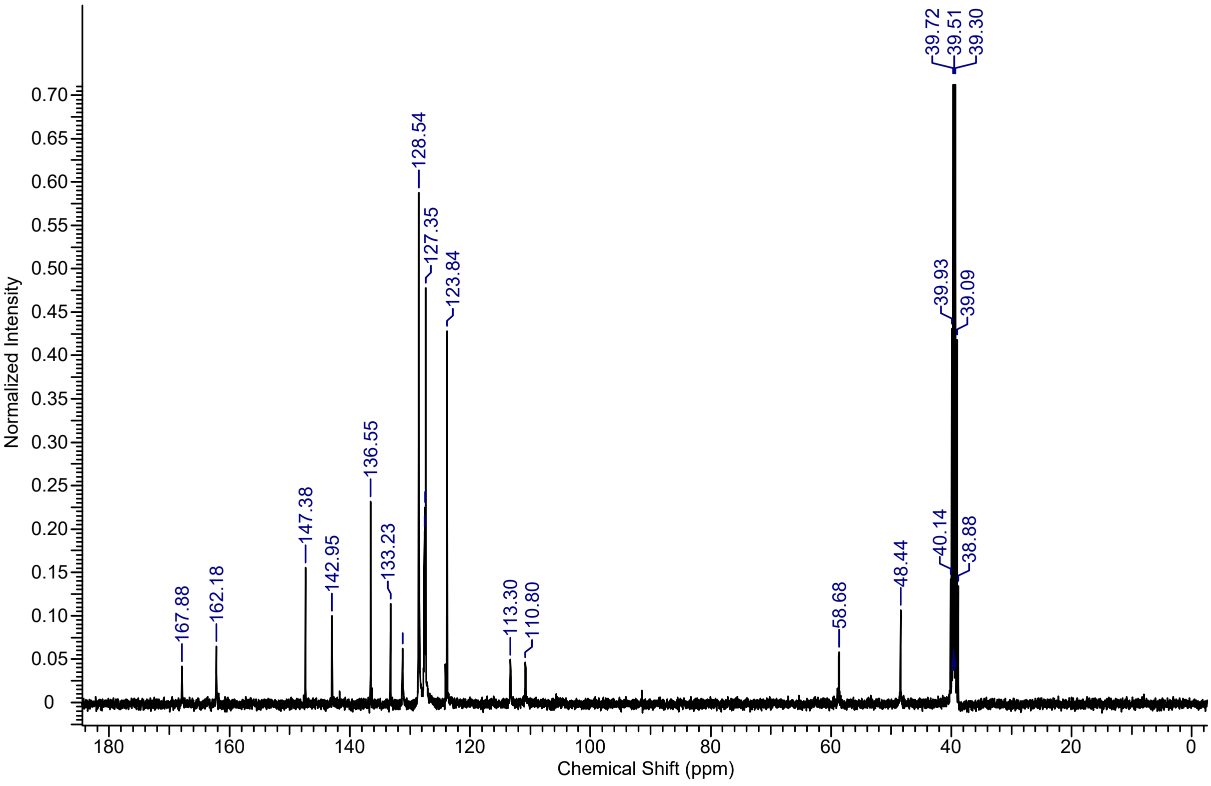

^{1}H and ^{13}C NMR spectra (d_6-DMSO) for compound 2b

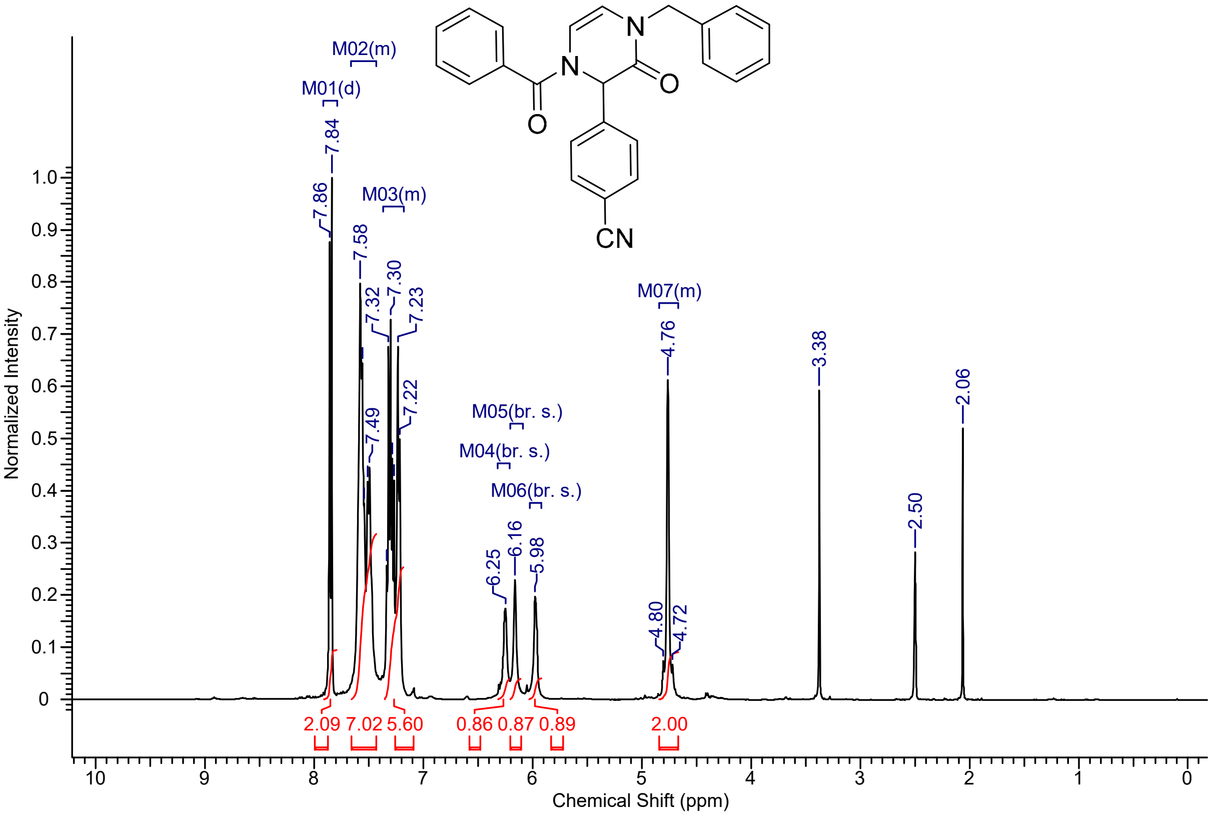

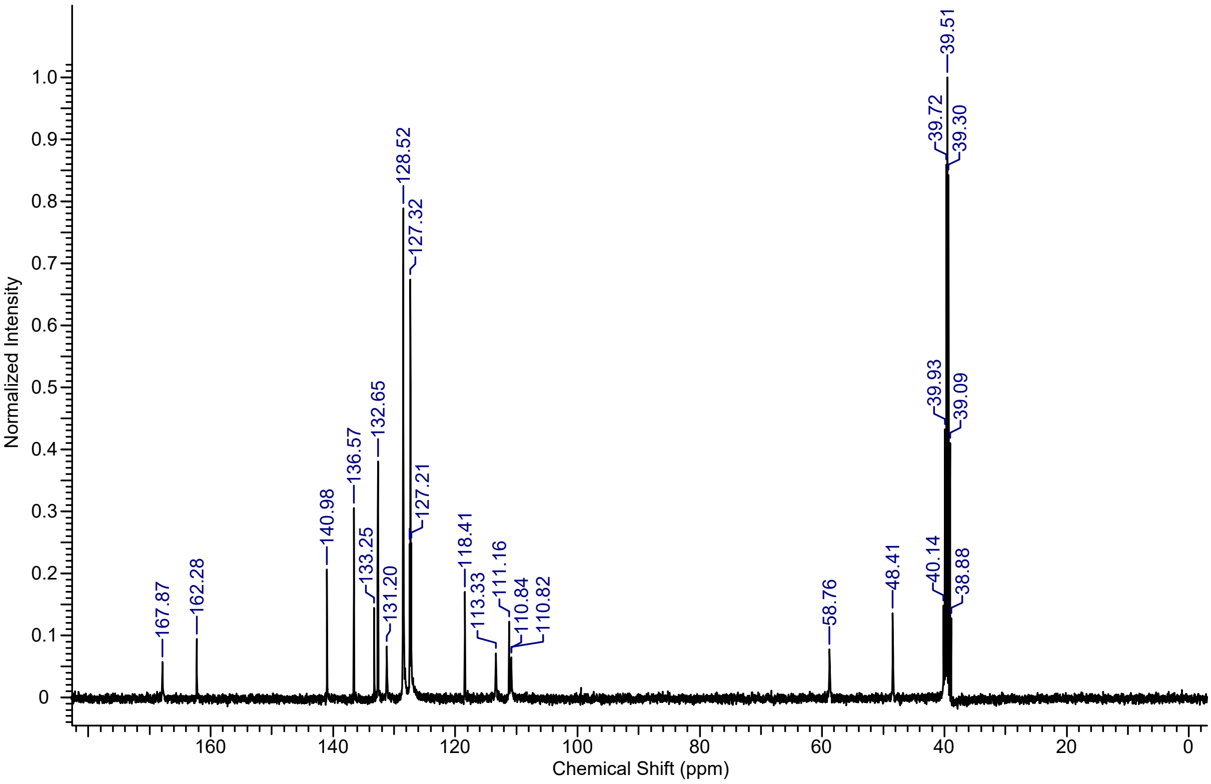

^{1}H and ^{13}C NMR spectra (d_6-DMSO) for compound 2c

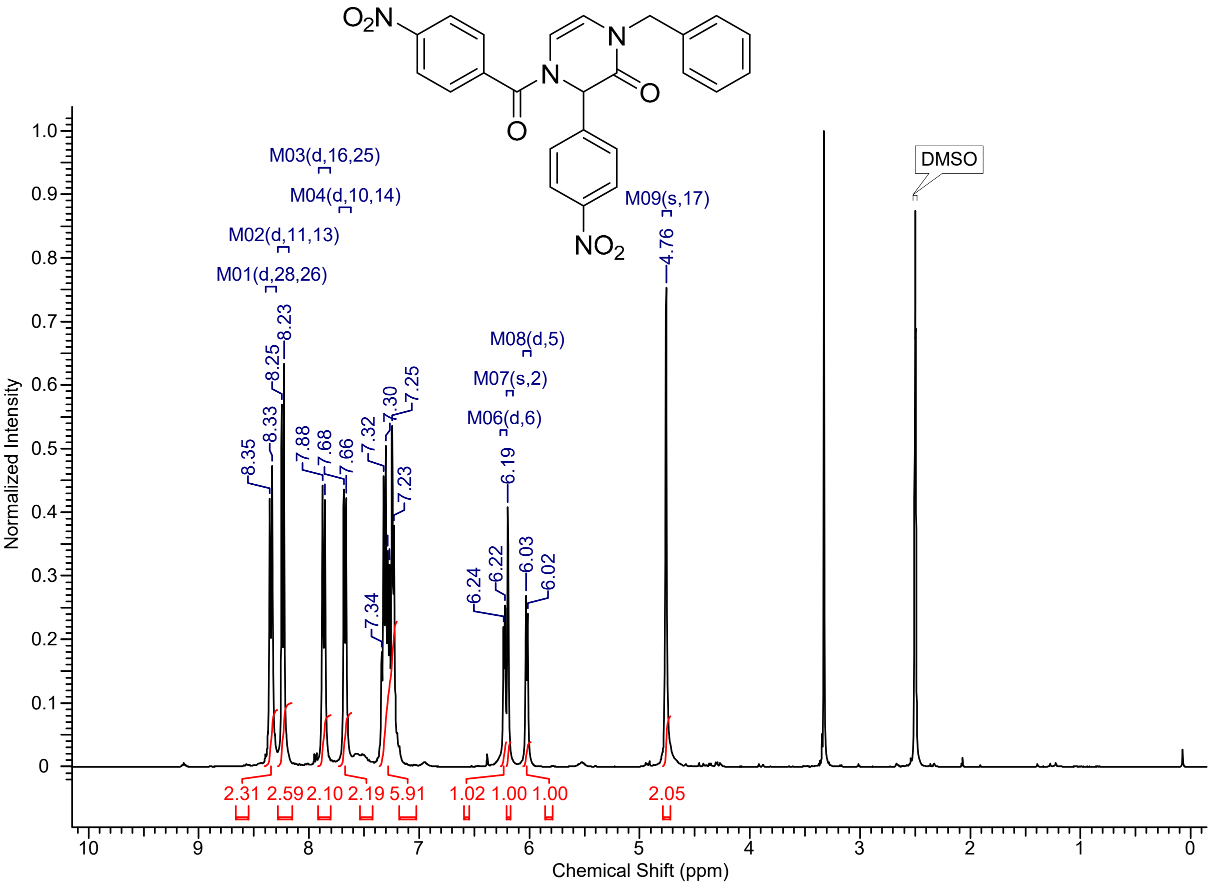

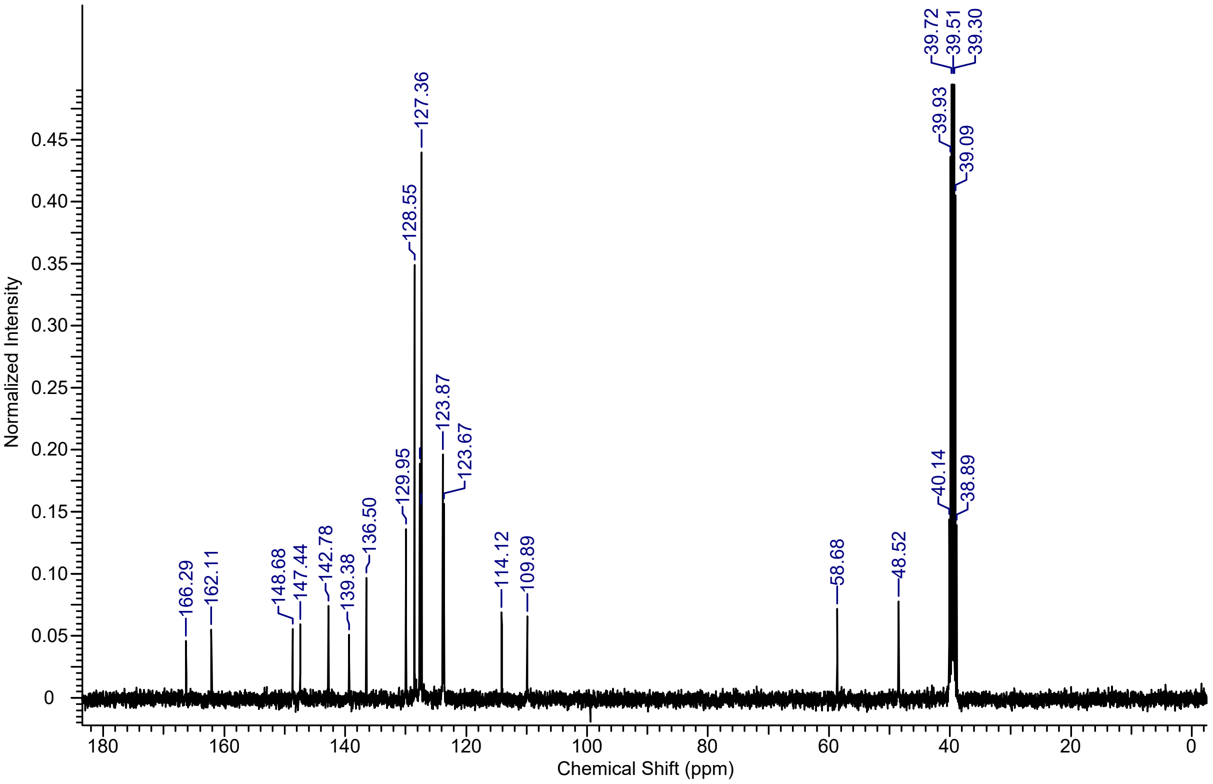

^{1}H and ^{13}C NMR spectra (d_6-DMSO) for compound 2d

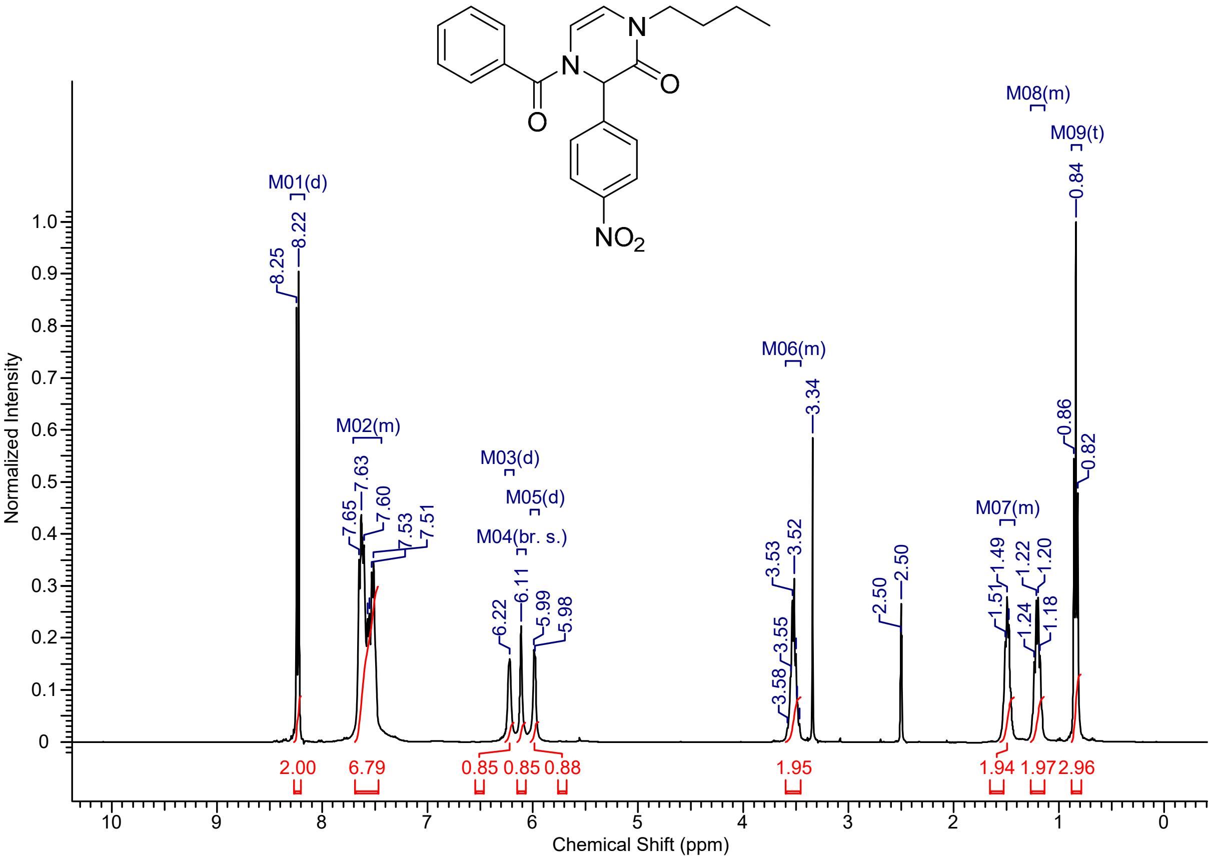

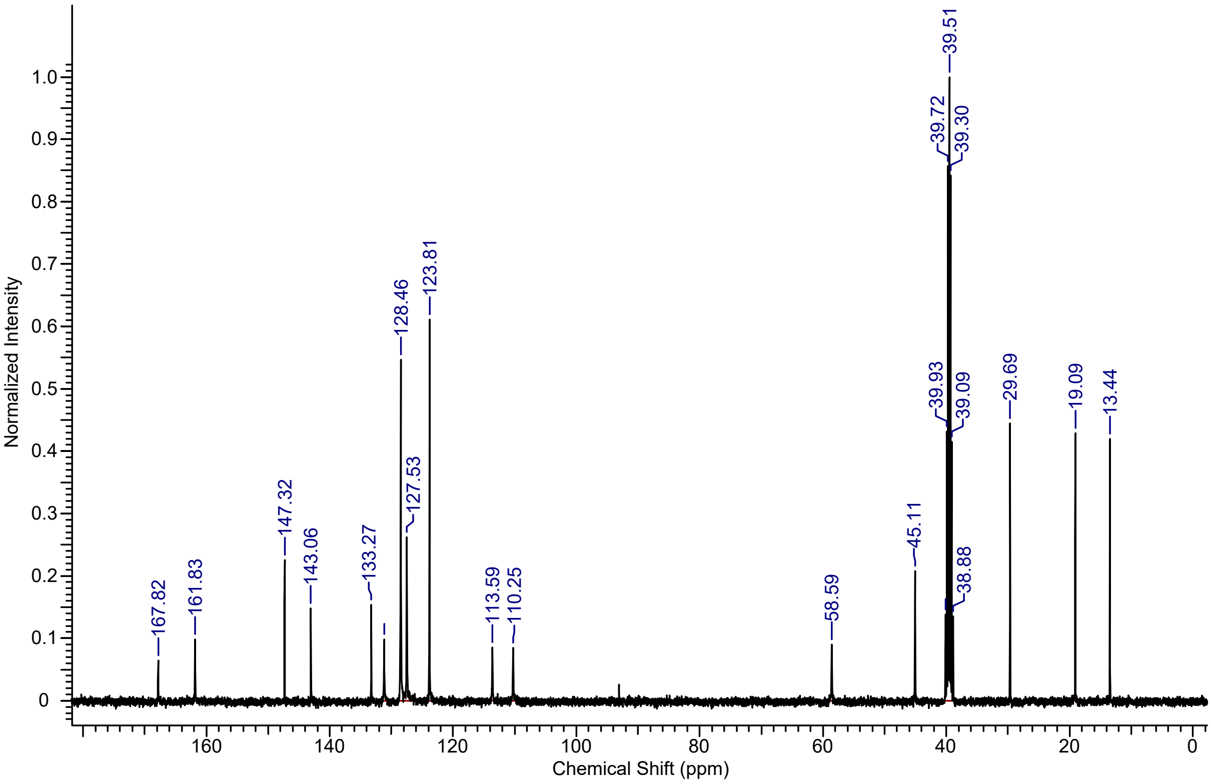

^{1}H and ^{13}C NMR spectra (d_6-DMSO) for compound 2e

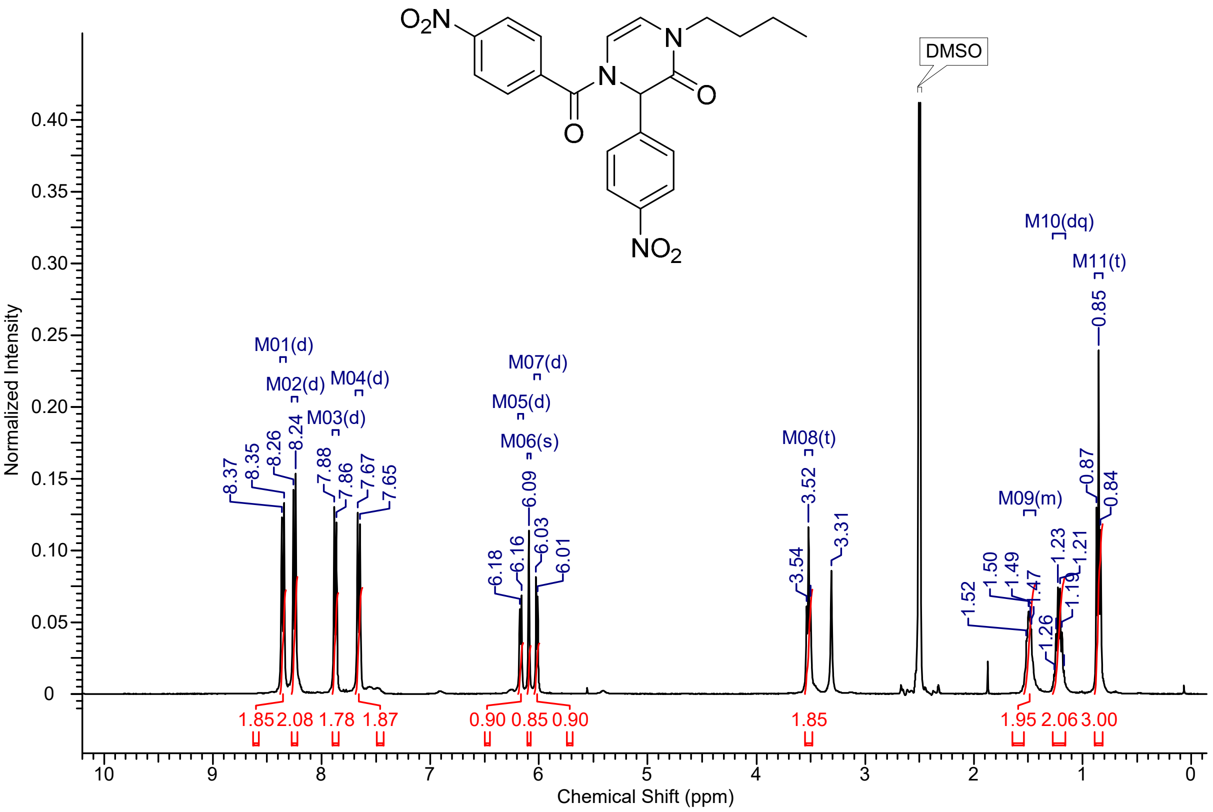

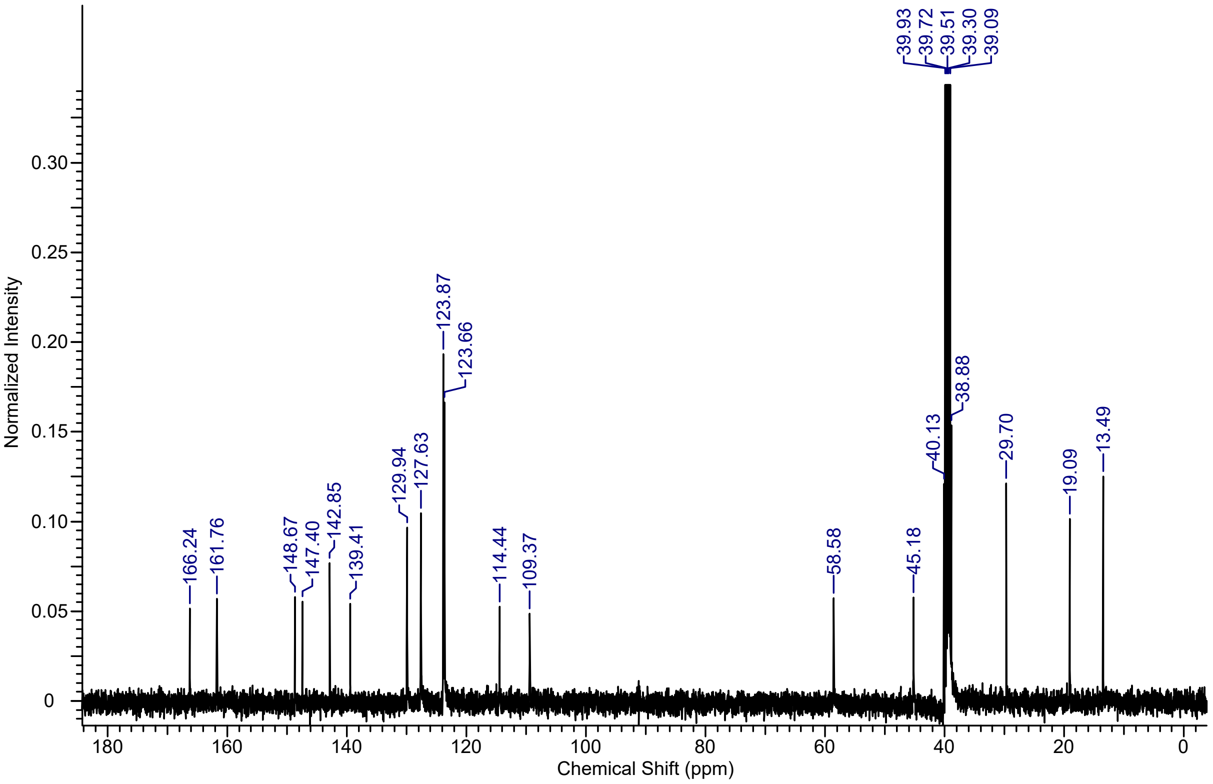

^{1}H and ^{13}C NMR spectra (d_6-DMSO) for compound 2f

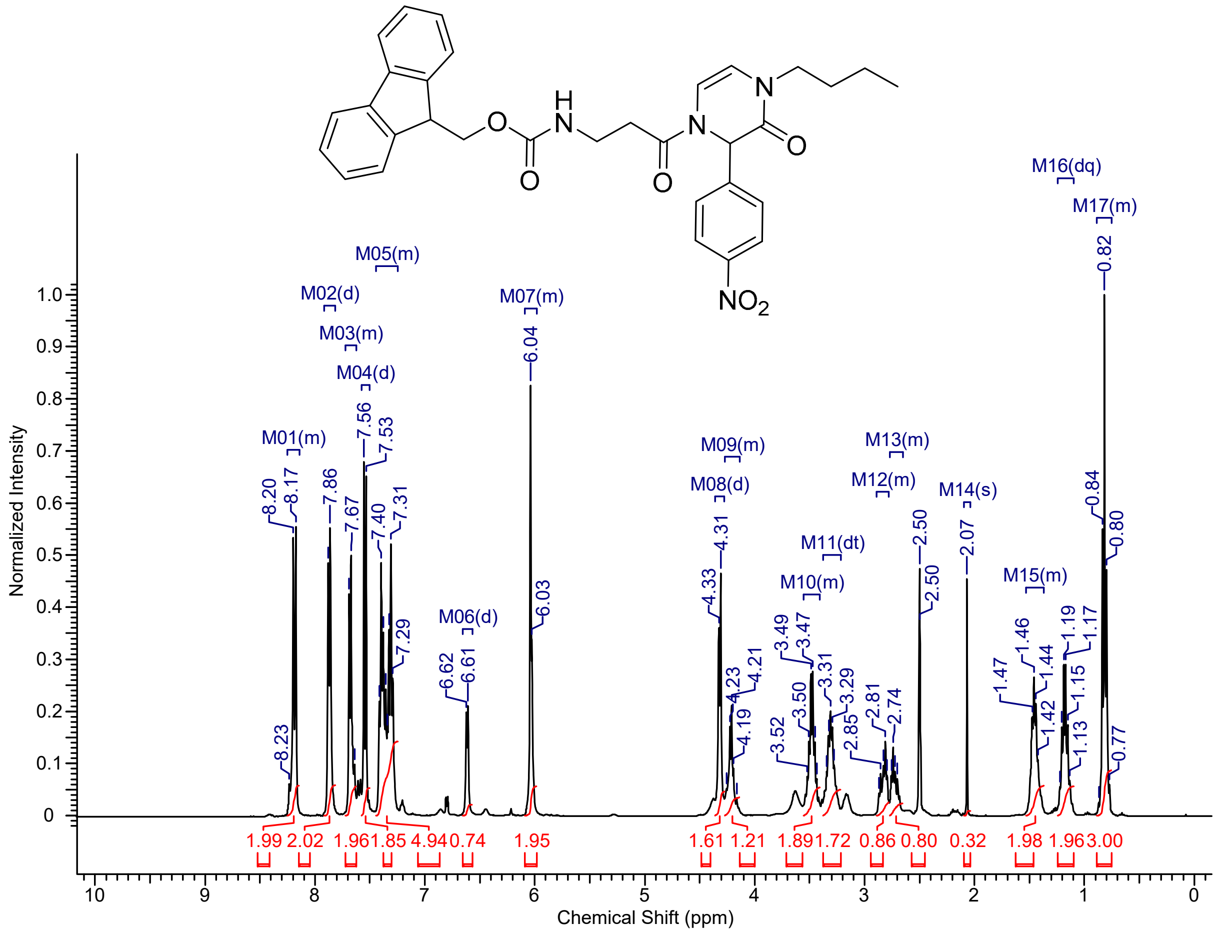

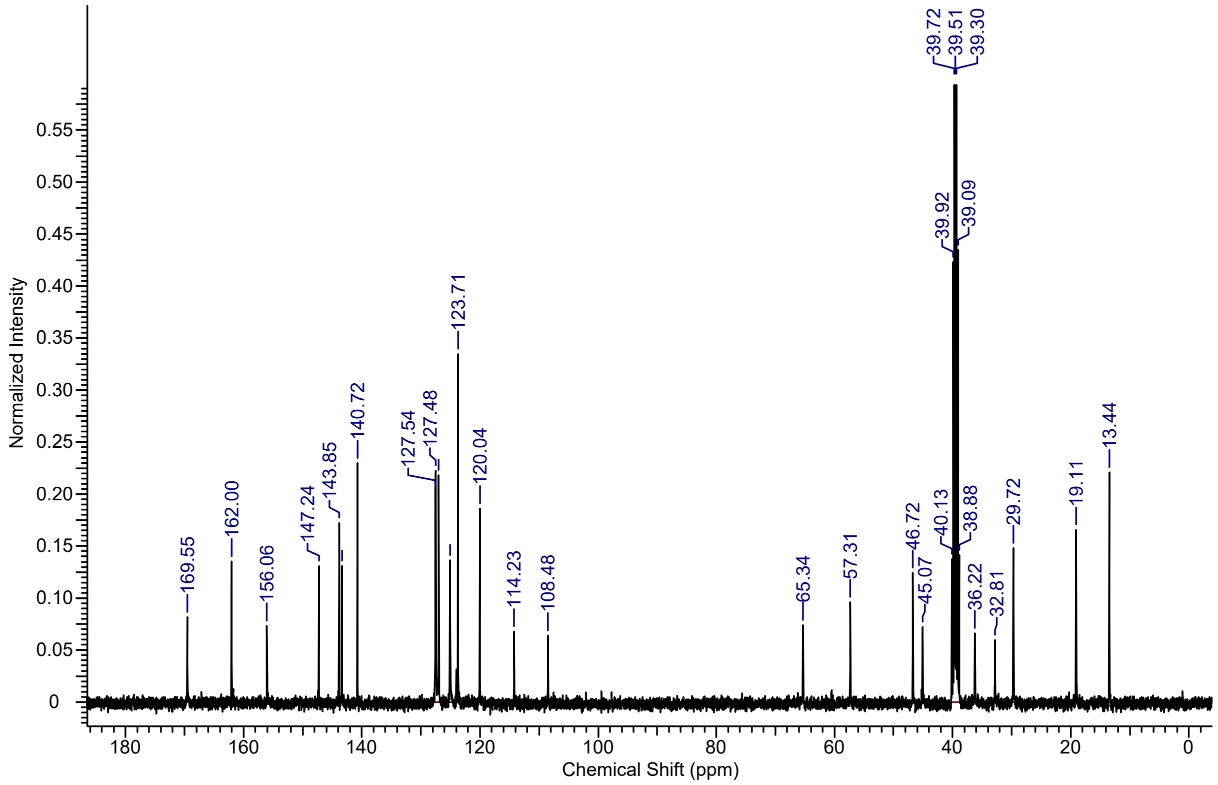

^{1}H – ^{1}H COSY spectrum (d_6-DMSO) for compound 2f

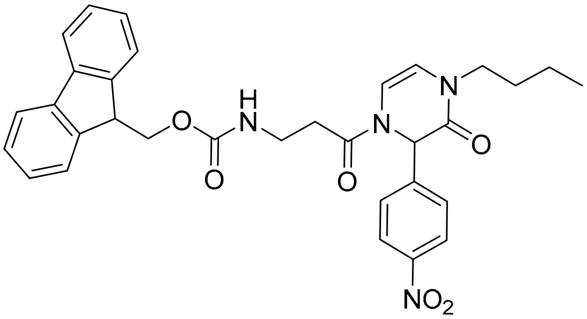

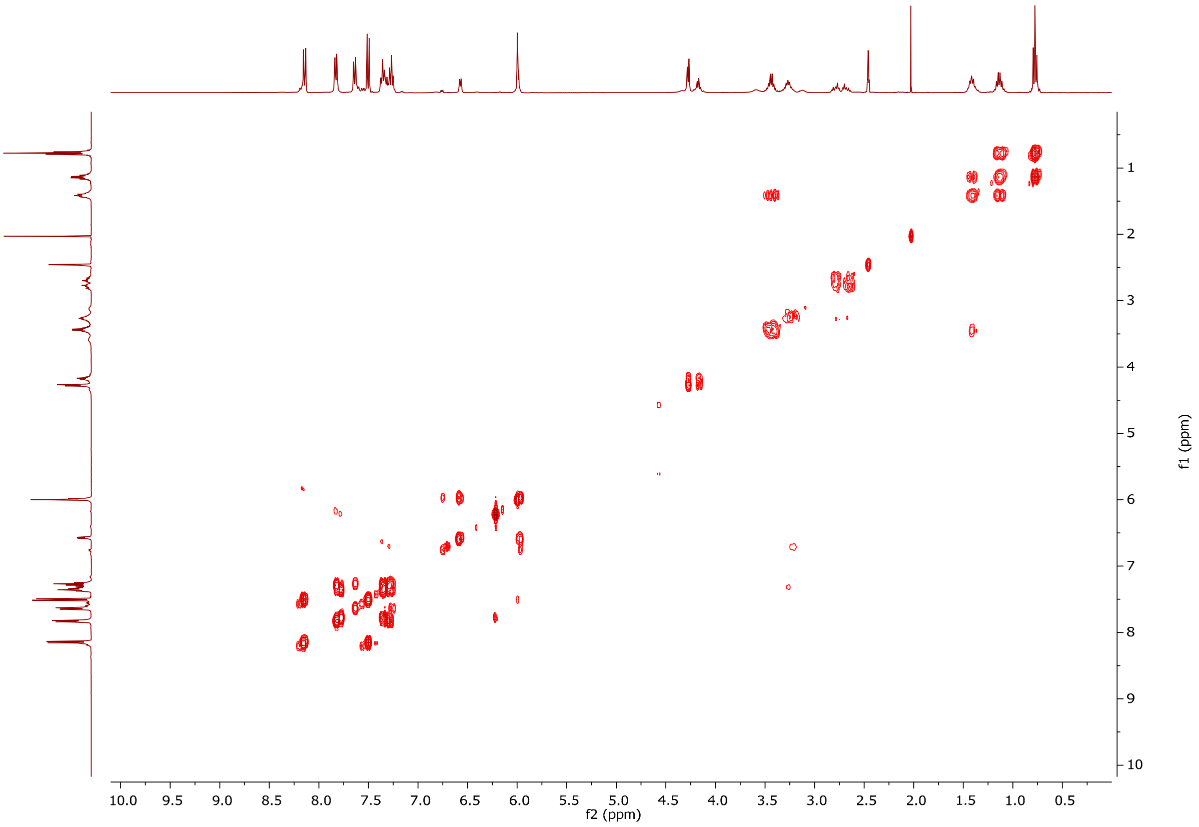

^{1}H and ^{13}C NMR spectra (d_6-DMSO) for compound 2g

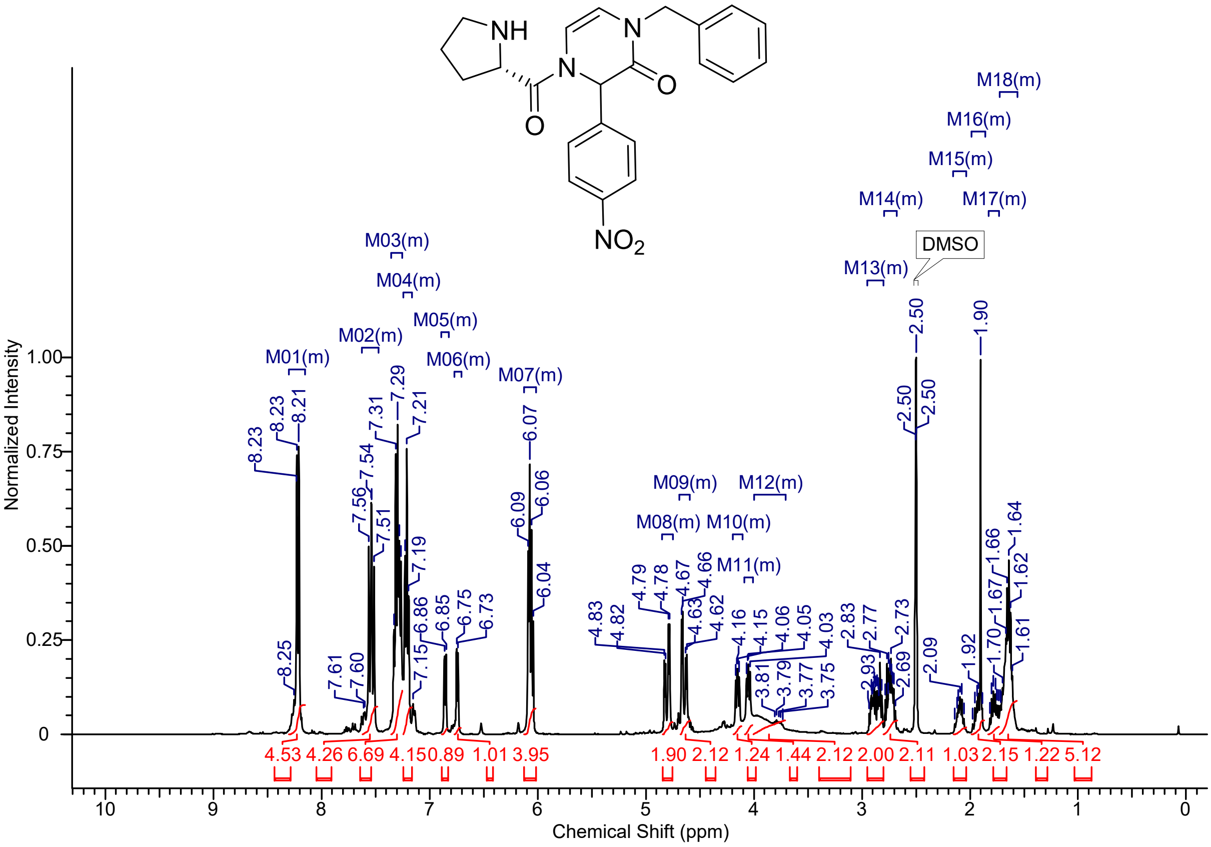

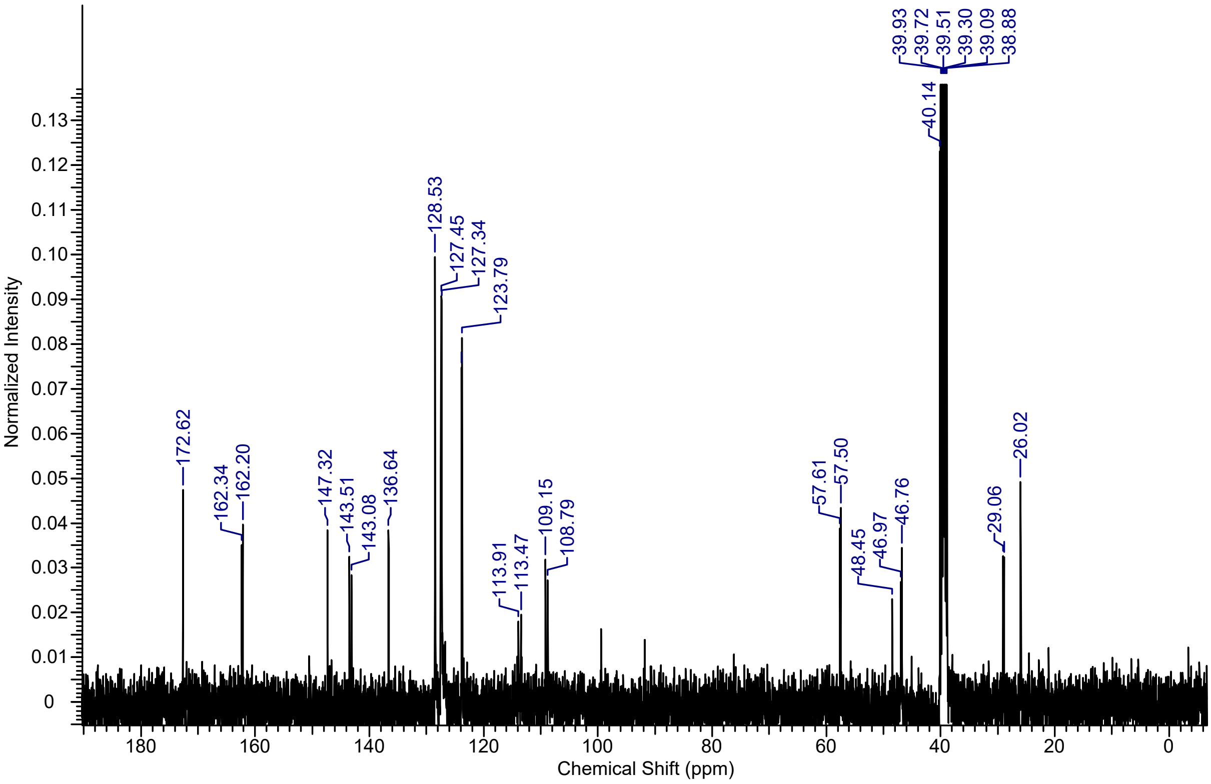

^{1}H – ^{1}H COSY spectrum (d_6-DMSO) for compound 2g

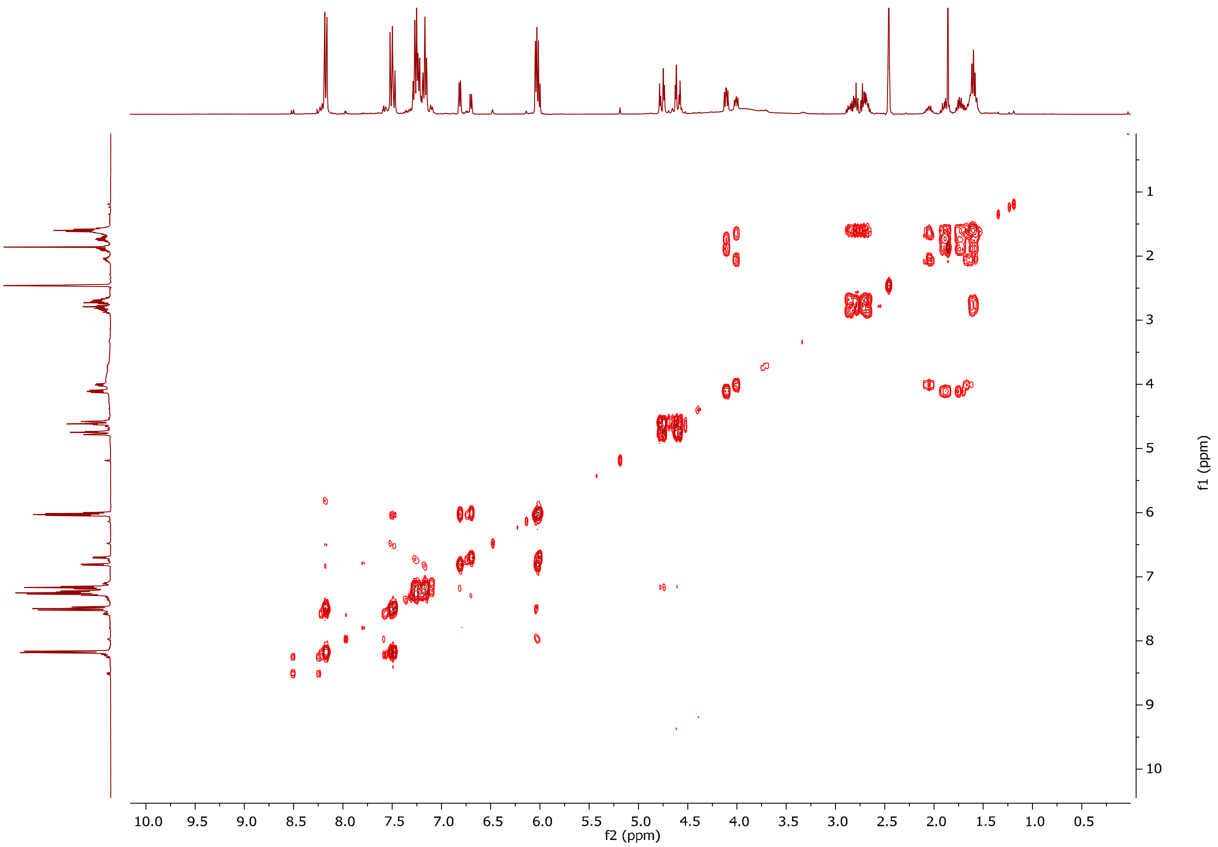

^{1}H – ^{13}C HMBC spectrum (d_6-DMSO) for compound 2g

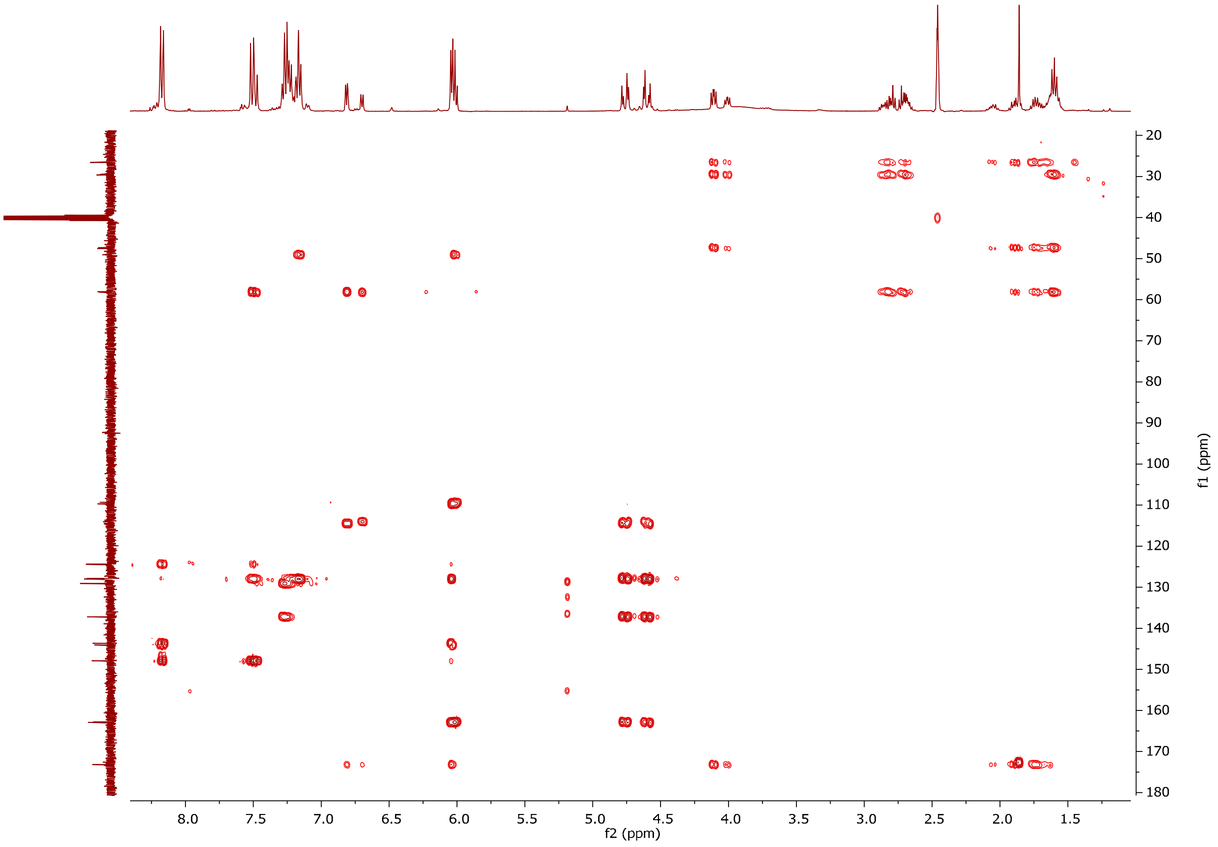

^{1}H and ^{13}C NMR spectra (d_6-DMSO) for compound 3a

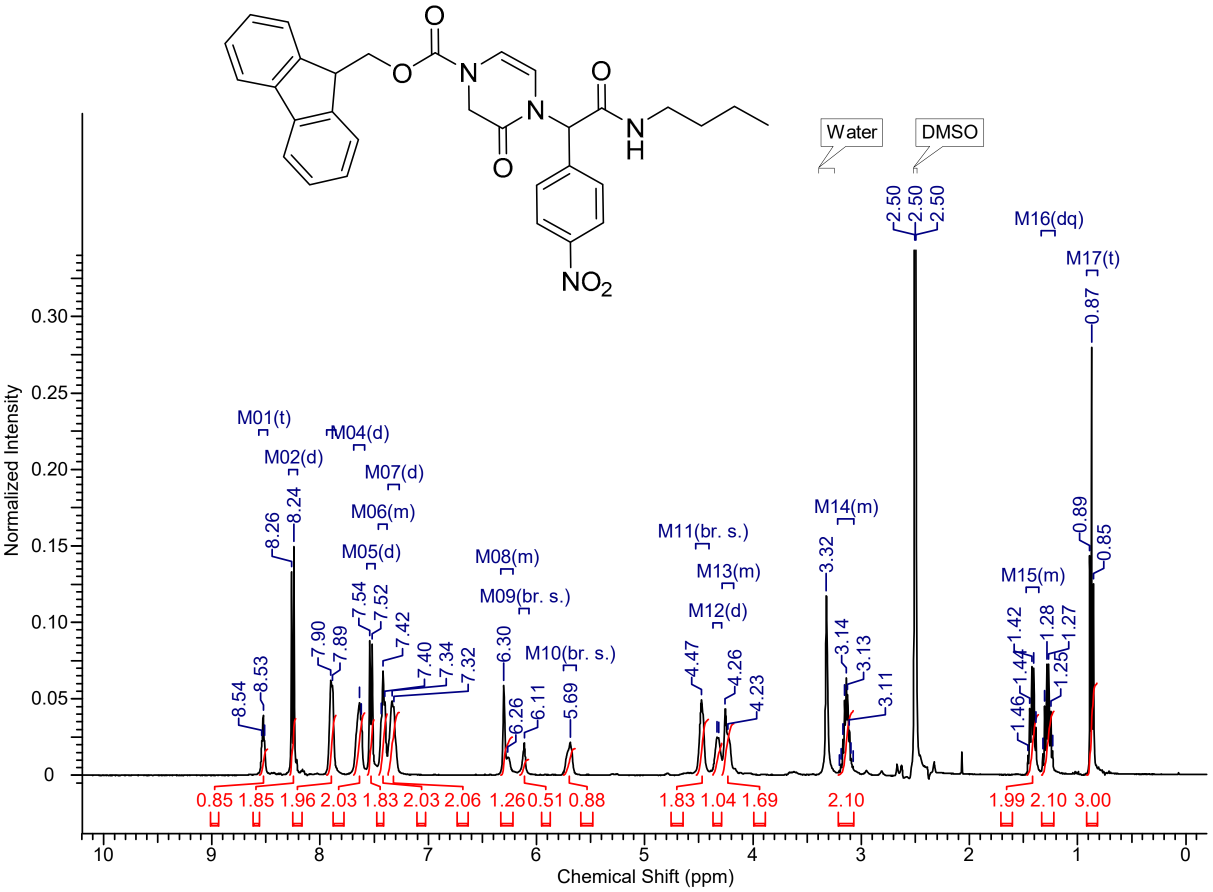

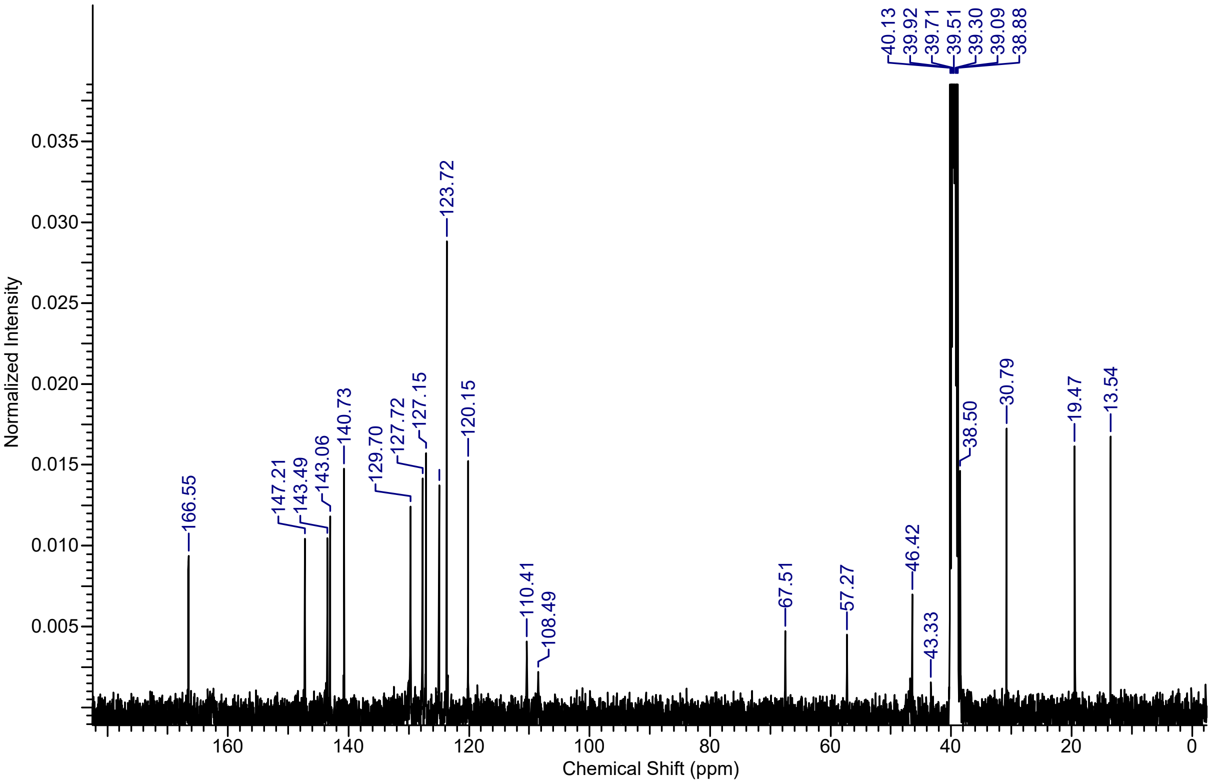

^{1}H and ^{13}C NMR spectra (d_6-DMSO) for compound 3b

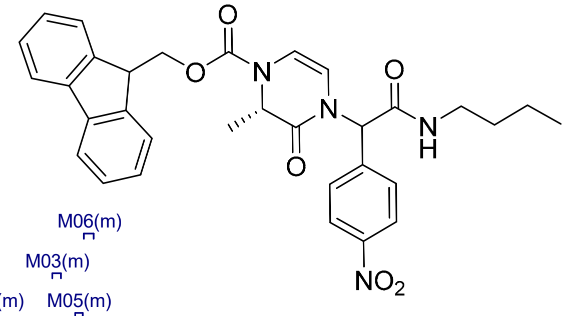

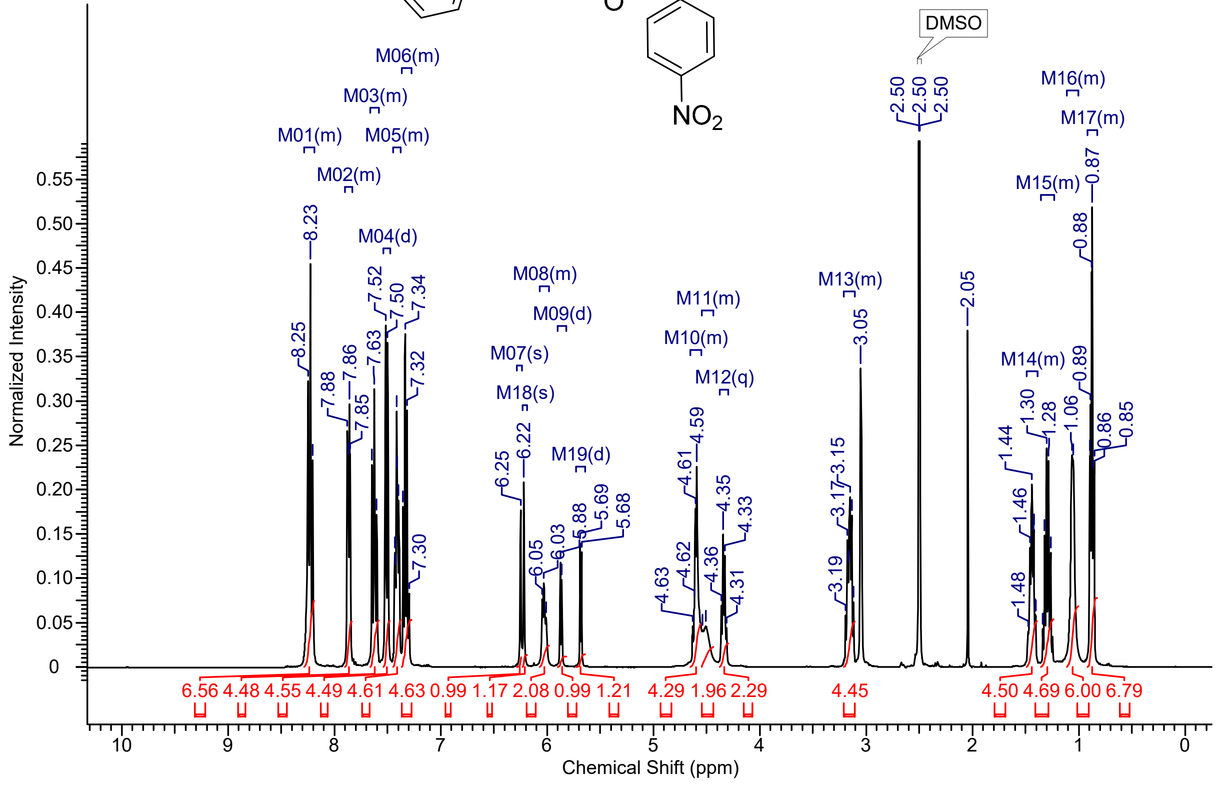

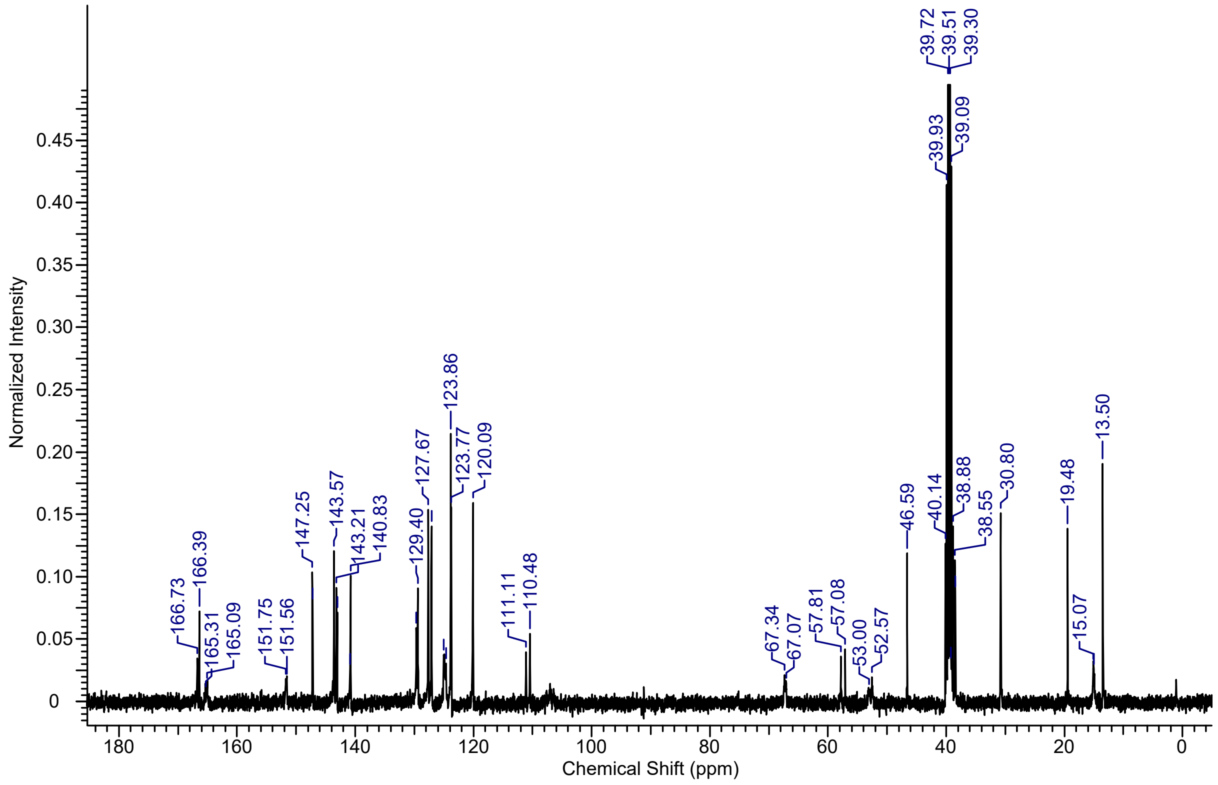

^{1}H and ^{13}C NMR spectra (d_6-DMSO) for compound 3c

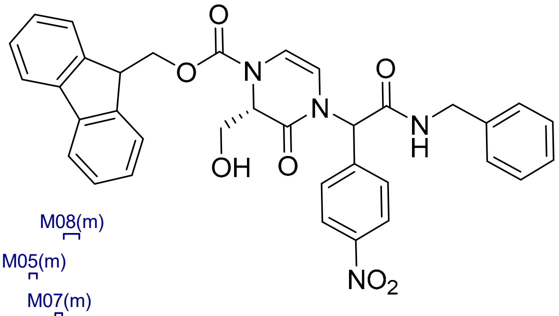

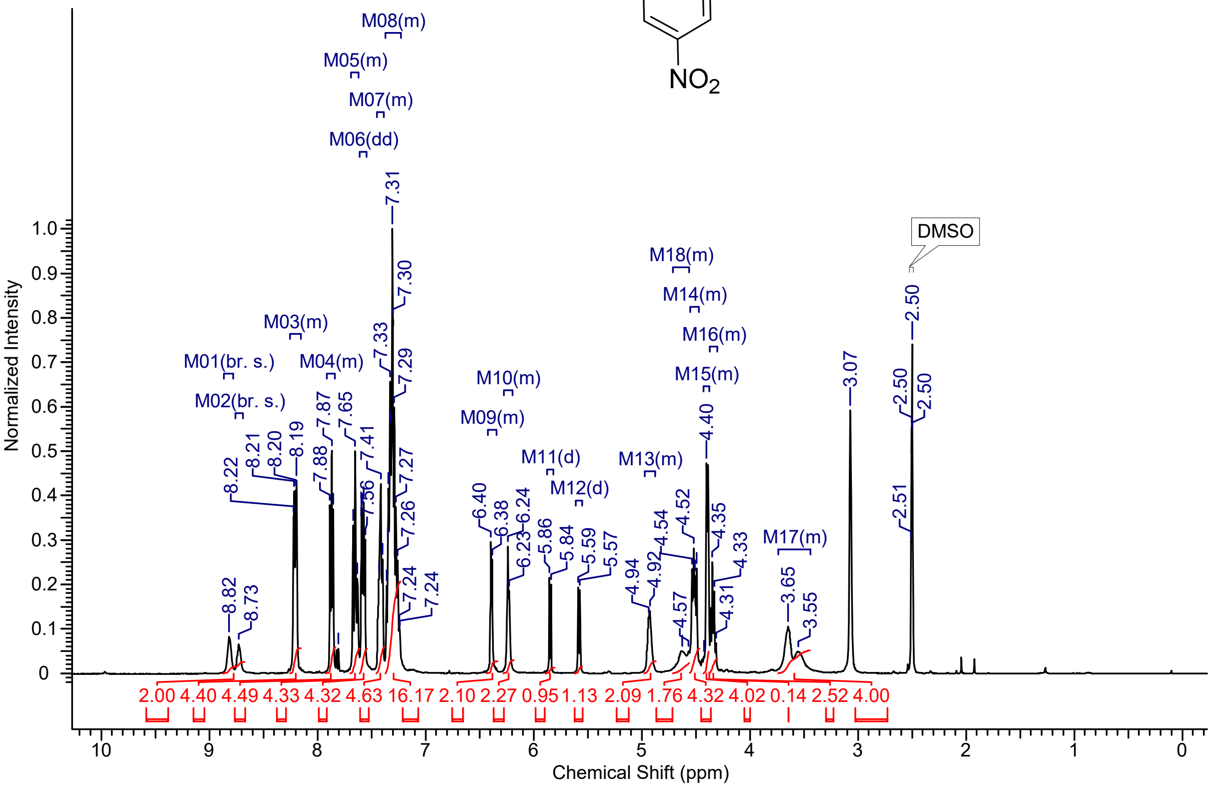

^{1}H and ^{13}C NMR spectra (d_6-DMSO) for compound 4a

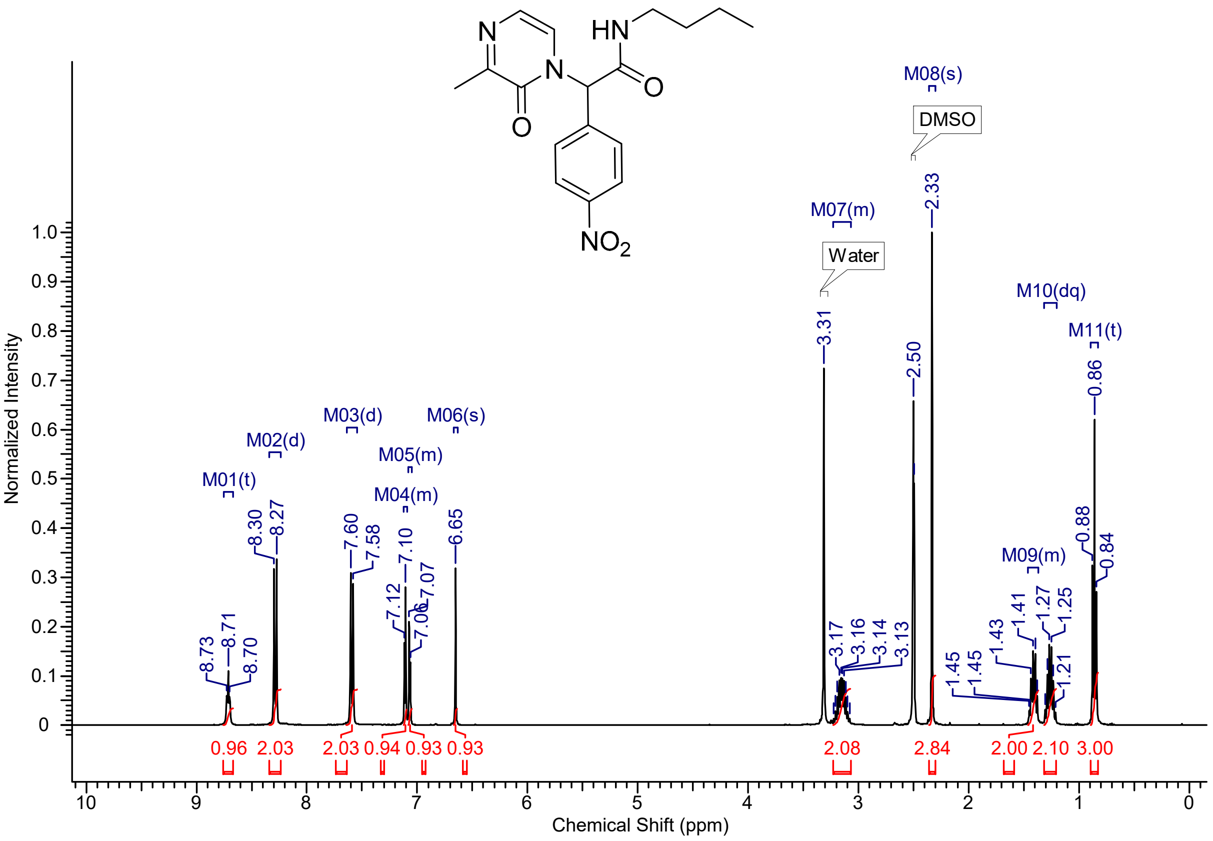

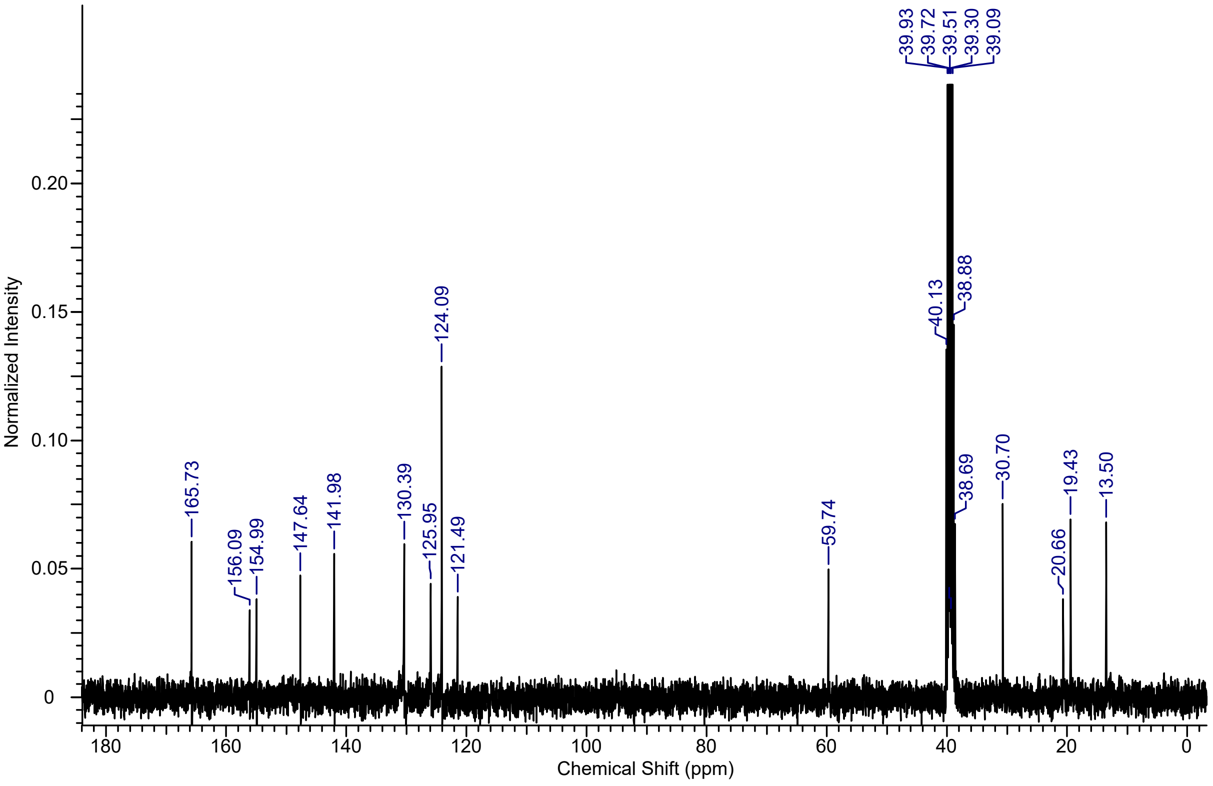

^{1}H and ^{13}C NMR spectra (d_6-DMSO) for compound 4b

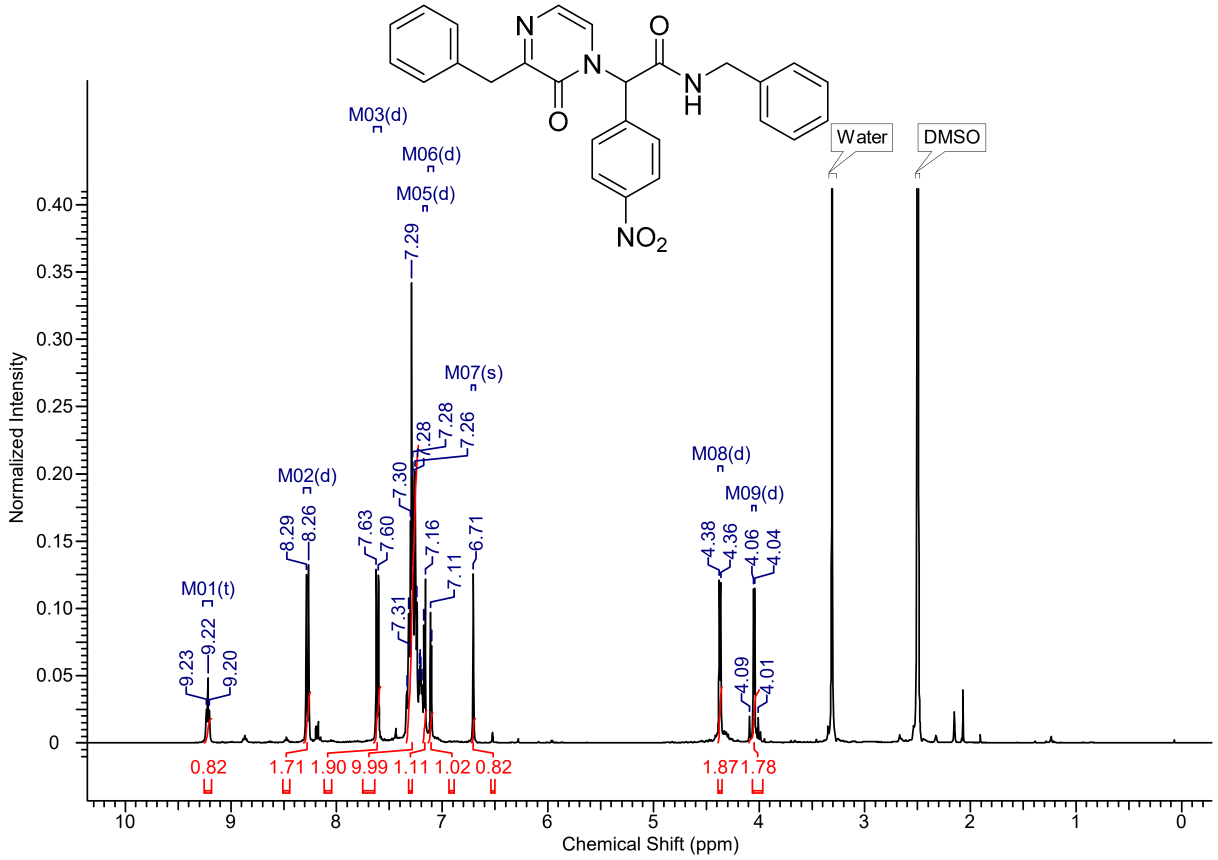

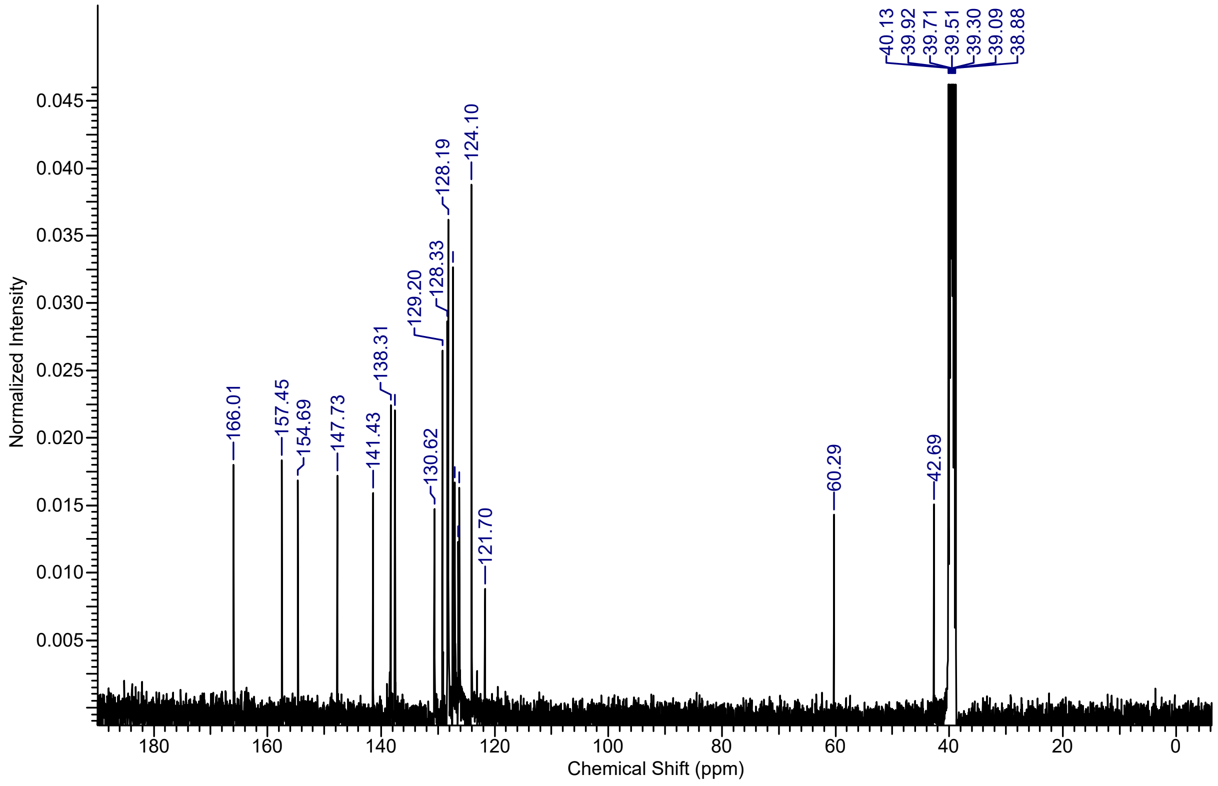

^{1}H and ^{13}C NMR spectra (*d_6*-DMSO) for compound 5a

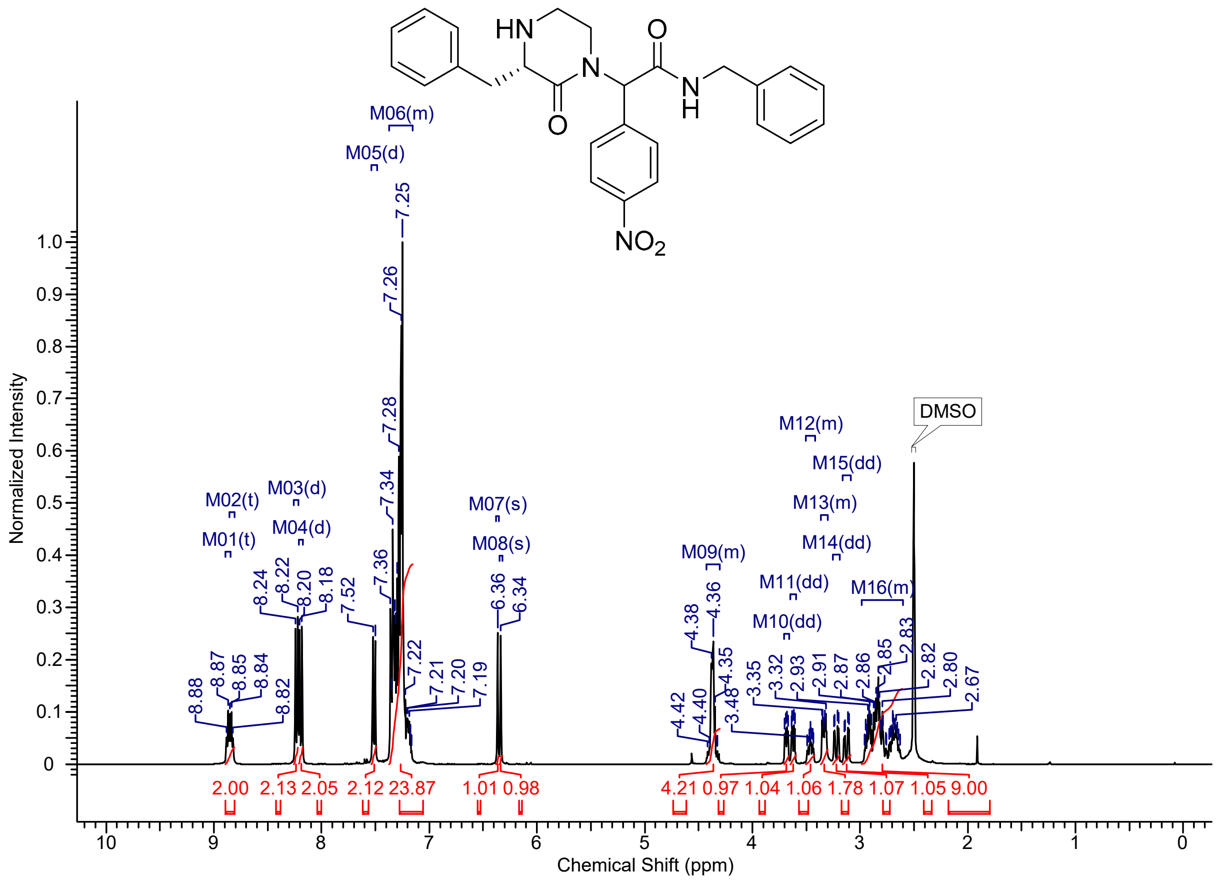

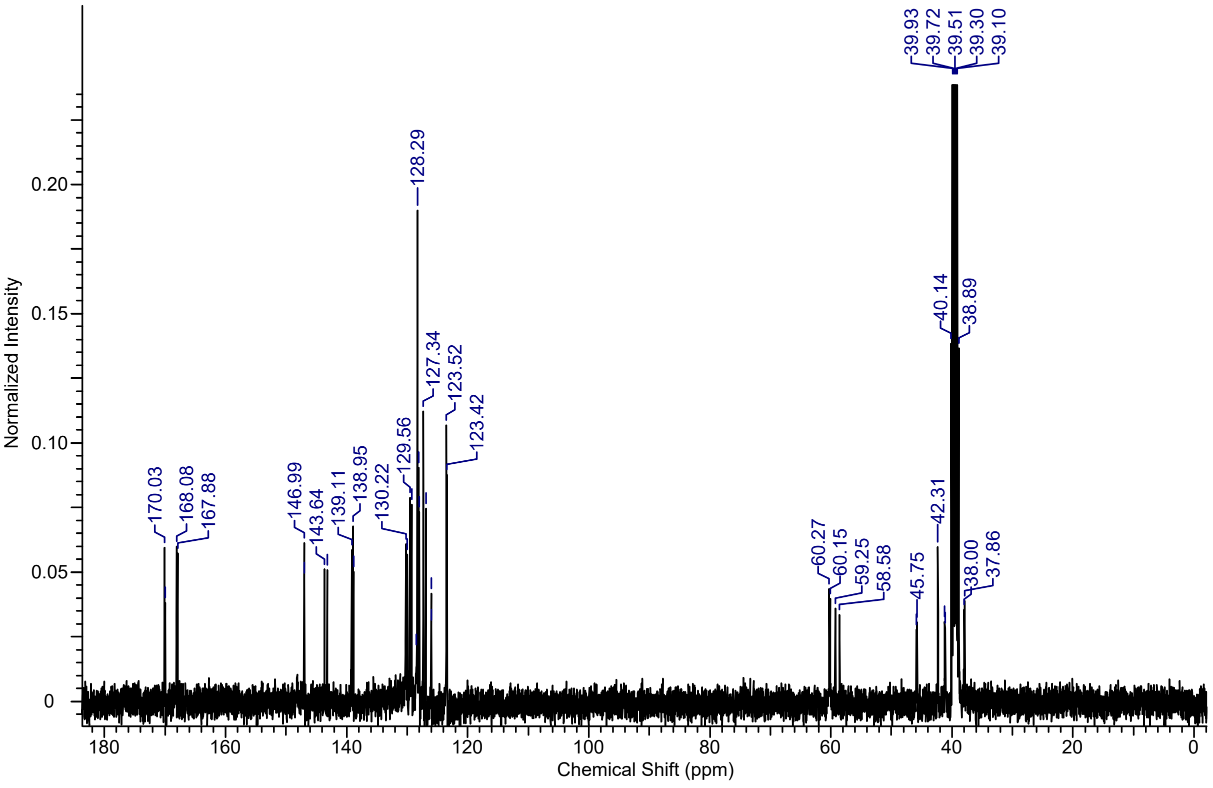

^{1}H and ^{13}C NMR spectra (d_6-DMSO) for compound 6a

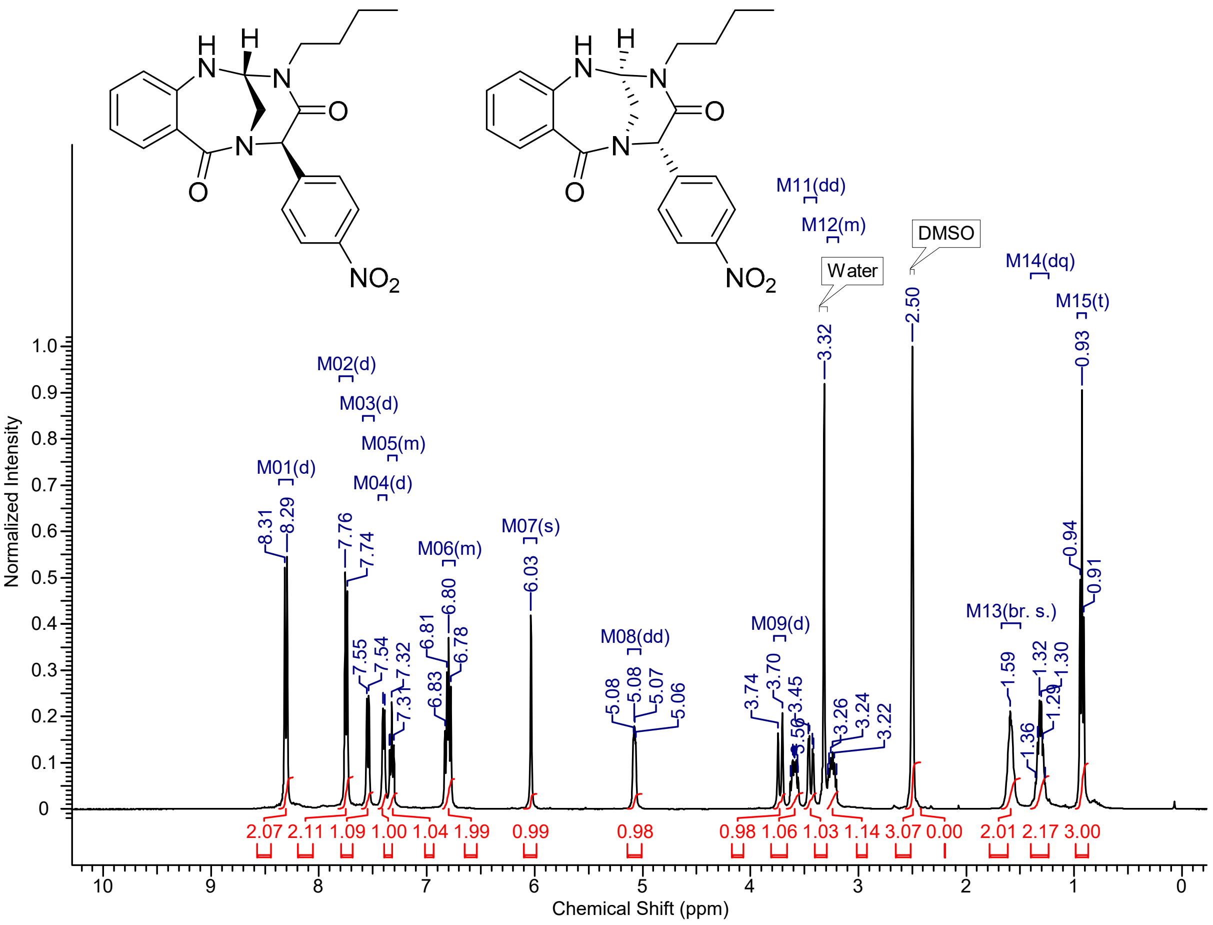

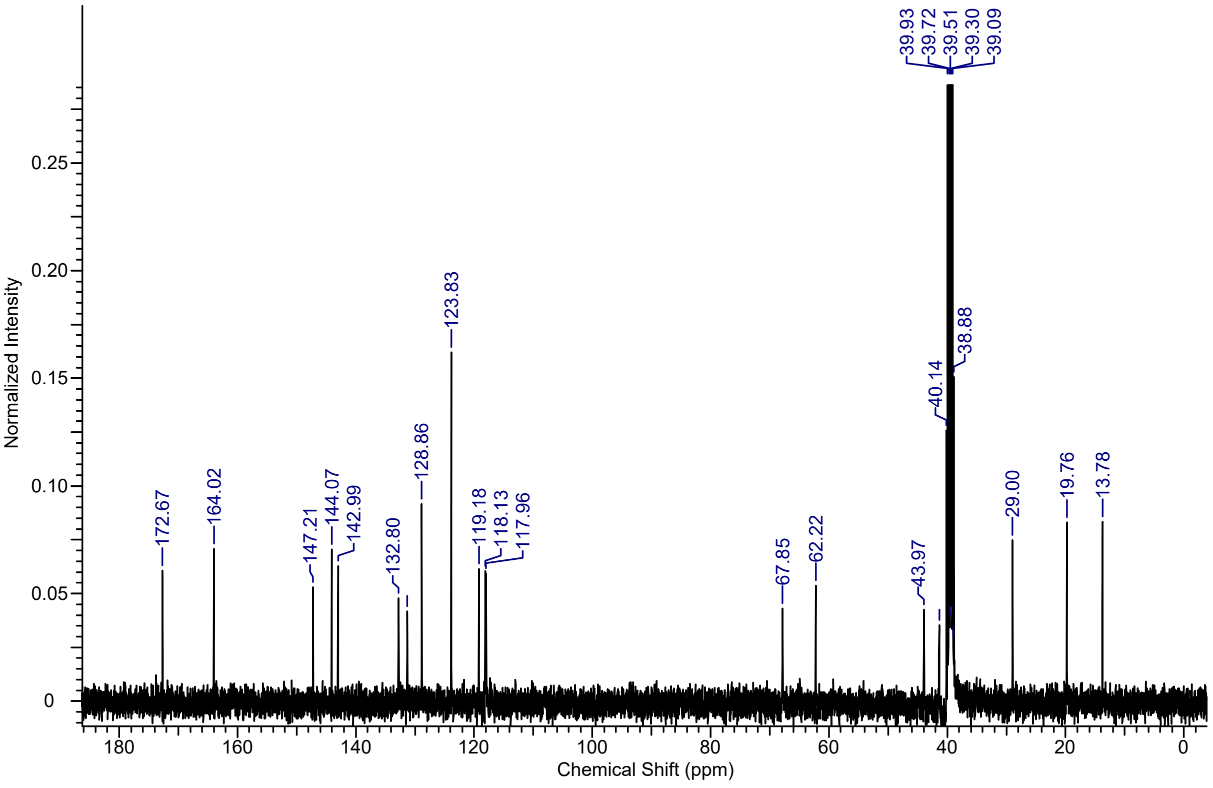

$^{1}H - ^{1}H$ COSY spectrum (d_6-DMSO) for compound 6a

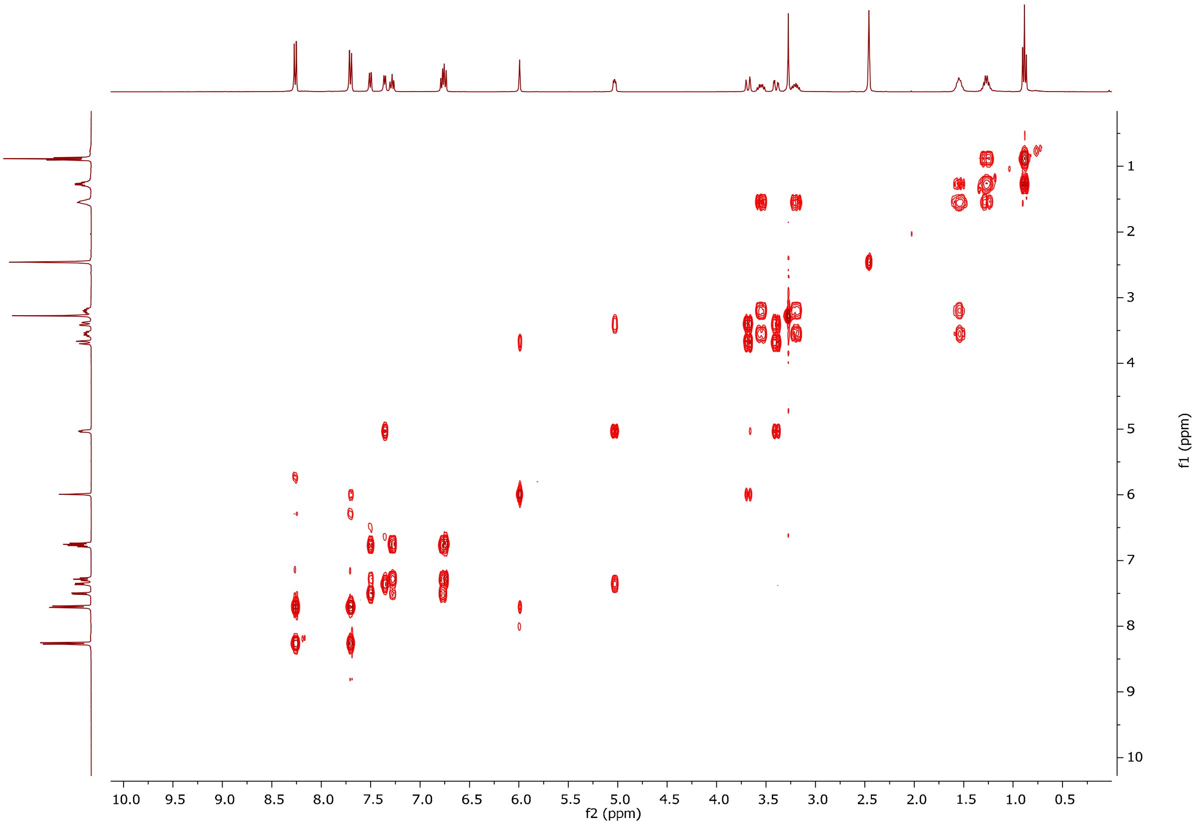

$^{1}H - ^{13}C$ HMBC spectrum (d_6-DMSO) for compound 6a

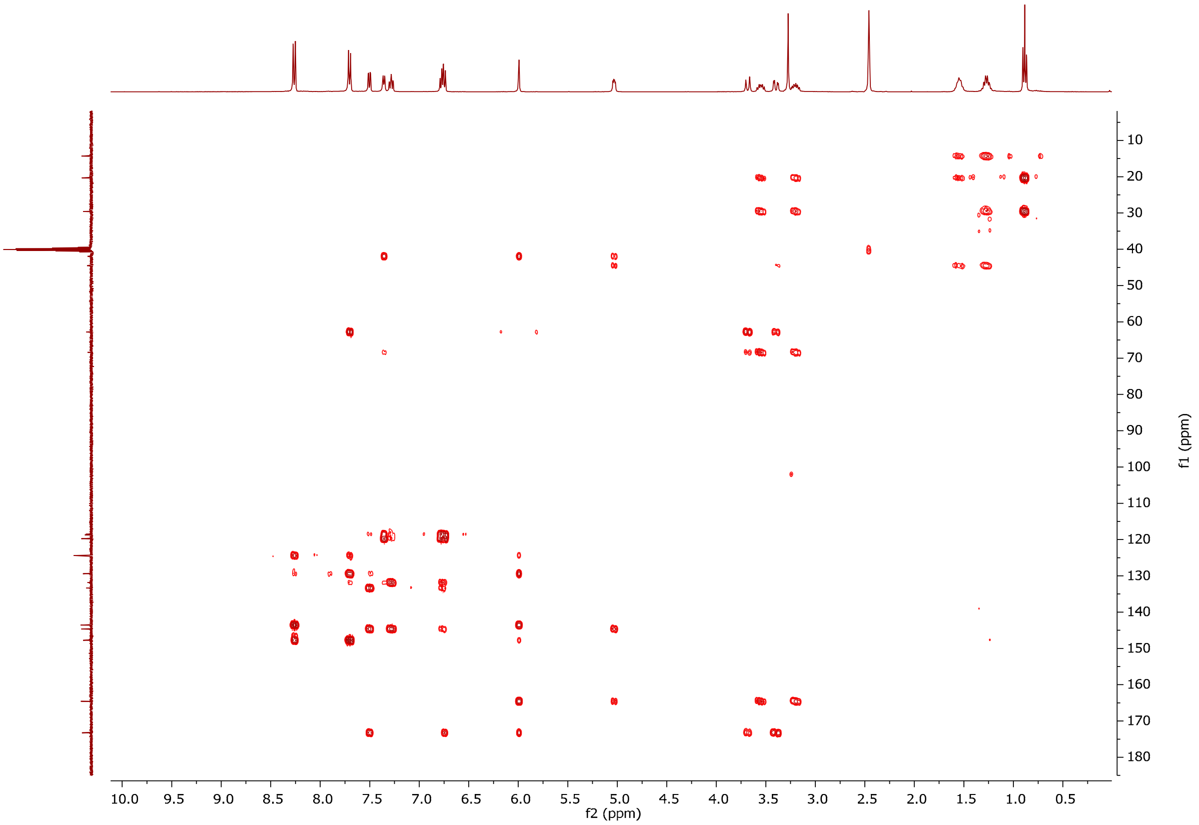

Supplement: Supplementary file 1 [file molecules-28-03062-s001.zip › molecules-2279789-supplementary.pdf]
